# Conformational-Dependent and Independent RNA Binding to the Fragile X Mental Retardation Protein

**DOI:** 10.4061/2011/246127

**Published:** 2011-05-29

**Authors:** Xin Yan, Robert B. Denman

**Affiliations:** ^1^CSI/IBR Center for Developmental Neuroscience, College of Staten Island, City University of New York, Staten Island, NY 10314, USA; ^2^Biochemical Molecular Neurobiology Laboratory, Department of Molecular Biology, New York State Institute for Basic Research in Developmental Disabilities, 1050 Forest Hill Road, Staten Island, NY 10314, USA

## Abstract

The interaction between the fragile X mental retardation protein (FMRP) and BC1 RNA has been the subject of controversy. We probed the parameters of RNA binding to FMRP in several ways. Nondenaturing agarose gel analysis showed that BC1 RNA transcripts produced by in vitro transcription contain a population of conformers, which can be modulated by preannealing. Accordingly, FMRP differentially binds to the annealed and unannealed conformer populations. Using partial RNase digestion, we demonstrate that annealed BC1 RNA contains a unique conformer that FMRP likely binds. We further demonstrate that this interaction is 100-fold weaker than that the binding of eEF-1A mRNA and FMRP, and that preannealing is not a general requirement for FMRP's interaction with RNA. In addition, binding does not require the N-terminal 204 amino acids of FMRP, methylated arginine residues and can be recapitulated by both fragile X paralogs. Altogether, our data continue to support a model in which BC1 RNA functions independently of FMRP.

## 1. Introduction

Fragile X syndrome is the most common inherited cause of mental impairment accounting for *ca.* 40% of X-linked mental retardation cases. It is also the most common known cause of autism (reviewed in [1–6]). Other characteristics of the fragile X syndrome include hyperactivity [7], increased susceptibility to seizures [8], increased testicular volume [9], macrocephaly, and large ears [10]. In addition, it has been found that carriers of the fragile X premutation, once thought to be free of the effects of the disease, also suffer from subtle behavioral and physical abnormalities [11–14]. This wide and varied constellation of phenotypic features results from the loss of function of a single gene, *FMR1* (summarized in: http://www.ncbi.nlm.nih.gov/bookshelf/br.fcgi?book=gene&part-fragilex). 

The *FMR1* gene encodes the RNA-binding protein FMRP [15], a negative [16–18] and positive [19, 20] translational regulator, and it has been of considerable interest to delineate the cellular RNAs that bind to FMRP [21–25] and the mechanism(s) by which FMRP binds and controls these mRNAs [26–35].

In 2003, Zalfa et al. described a bridging mechanism in which the fragile X mental retardation protein (FMRP) *via *interaction with the 5′ end of the small noncoding RNA, BC1, and bound and repressed FMRP target mRNAs [36]. This model has been subject to great deal of scrutiny owing to findings that appear to be out of step with other studies. These include differences in the prime localization of FMRP with small repressed mRNPs rather than brain polyribosomes [18, 37–39], differences in the interpretation of the interaction of FMRP with BC1 RNA as specific and significant [40] rather than nonspecific and insignificant [30, 41, 42] and detailed mechanistic differences in the nature of BC1 RNA-mediated localization and translational repression [41, 43–45]. In response to some of these criticisms, Zalfa and Bagni reposited that their model, rather than being a *general* model of FMRP-mRNA interactions, was only one of several possible models [46].

More recent investigations of some of the concomitants of the Zalfa model [47] found that the interaction of recombinant FMRP with BC1 RNA was weak compared to that of a G-quartet-containing RNA. Furthermore, its strength varied significantly depending upon the buffer conditions used. The data suggested that FMRP may interact with a particular conformer of BC1 RNA. Here, we elaborate the conditions and requirements for a weak FMRP BC1 RNA in vitro interaction.

## 2. Materials and Methods

### 2.1. Buffers

Z-buffer is 10 mM Tris-HCl pH 7.5, 2 mM MgCl_2_, 400 mM NaCl and 0.2% SDS [36]. The RNA-binding buffer that was used for affinity capture is 50 mM Tris-HCl, pH 7.4, 2 mM MgCl_2_, and 150 mM KCl, 1 mM EDTA, and 1 mM DTT [26]. Structure buffer is 10 mM Tris-HCl pH 7, 100 mM KCl, and 10 mM MgCl_2_.

### 2.2. Preparation of BC1 RNA Transcripts

*Dra I* linearized pBCX607 containing the entire BC1 sequence [48], *AvaII* linearized pBCX607 containing the first 65 b of BC1, *Sac I* linearized pMK-1 containing the last 60 b of BC1 RNA, a PCR fragment encoding a T7 RNA polymerase promoter and the first 75 b of BC1 RNA, *Hind III* linearized pTAR encoding an 85 b transcript that folds into a 57 b TAR element and a 28 b leader sequence and linearized pTri-XEF1 encoding eEF-1A mRNA (Ambion) were used to produce biotinylated RNAs via *in vitro *transcription (Ambion). Plasmids pBCX607 and pMK-1 were provided by Dr. Henri Tiedge (SUNY Brooklyn). Alcohol-precipitated RNAs were dissolved in 50 *μ*L DEPC-treated H_2_O and quantified spectrophotometrically. RNA integrity was examined by agarose gel electrophoresis. For examining the effect of annealing on RNA-protein interactions, individual RNAs were heated in 1x transcription buffer (Ambion) at 65°C for 10 min and then allowed to cool slowly to room temperature for one hour prior to their use. Note. We obtained identical results using Z-buffer in the annealing reaction (not shown).

### 2.3. RNA-Binding Assays

^35^S-FMRP, ^35^S-FMRP_280_, ^35^S-FMRP_204_, ^35^S-FXR1P, ^35^S-FXR2P, ^35^S-eIF4A, and ^35^S-luciferase and were produced from plasmids pET21A-FMRP, pND-L-mHisFMRP, pET9-FMRP_280_, pET9-FMRP_204_, pHA-FXR1P, and pET21b-FXR2P in an RRL-coupled transcription-translation system (Promega). Plasmid pHA-FXR1P was provided by Dr. Gideon Dreyfuss (University of Pennsylvania); plasmids pET9-FMRP_280_ and pET9-FMRP_204_ were provided by Dr. Darryl Spinner (IBR), plasmid pET21b-FXR2P was a gift from Dr. Jennifer Darnell (Rockefeller University) and plasmid pET-His6-eIF4A was a gift from Dr. Henri Tiedge (SUNY Brooklyn). Briefly, ^35^S-labeled- proteins were produced by combining twenty five microliters of TNT rabbit reticulocyte lysate (RRL) with 2 *μ*L of TNT T7 RNA polymerase, 1 *μ*L of 1 mM amino acid mix minus-methionine, 35 *μ*Ci of ^35^S-methionine, 1 *μ*L of RNasin and 1 *μ*g of plasmid DNA in a total volume of 50 *μ*L. One microliter of a 50X Complete protease inhibitor cocktail was added to prevent proteolysis. Samples were incubated at 30°C for 90 min and then assayed for protein production by autoradiography.

Affinity capture assays were performed as described previously [17]; the bound and unbound products were resolved by SDS-PAGE and subject to autoradiography. 

Autoradiograms were quantified using UN-SCAN-IT Gel 6.1 (Silk Scientific, Inc.). The percent binding was calculated as

(1) $$\text{\%Binding}=100\times \frac{{\text{Intensity}}_{\text{bound}}}{\left[{\text{Intensity}}_{\text{bound}}+{\text{Intensity}}_{\text{unbound}}\right]}.$$

The percent binding of the “no RNA” control in each experimental set was subtracted from that of the samples; the difference, representing authentic binding, was plotted.

### 2.4. RNA Structure Studies

Annealed and unannealed BC1 RNA, BC1 fragment RNAs and control RNAs (1-2 *μ*g), were treated at room temperature for 15 min with various amounts of ribonuclease V1 (cobra venom) or ribonuclease A in structure buffer as indicated. Reaction products were resolved on 1-2% TAE agarose gels containing 0.1 *μ*g/mL ethidium bromide along with appropriate size markers. Gels were imaged using a Scion CFW-1308 M mega pixel camera and captured in inverted mode using FOTO/Analyst PC Image software version 9.04 (FOTODYNE). The resulting image files were digitized and analyzed using UN-SCAN-IT Gel 6.1.

### 2.5. RNA Secondary Structure Modeling

RNA lowest energy secondary structures were determined using the Zuker algorithm, M-fold (http://mfold.rna.albany.edu).

## 3. Results and Discussion

### 3.1. Affinity Capture of FMRP with Biotinylated RNA

Several methods have been used to assess the direct physical interaction of FMRP with RNA *in vitro*. These include pull-down assays with homoribopolymers [15, 21, 49, 50], affinity capture using biotinylated RNA [21, 41, 51–53], affinity capture using immobilized protein [16], UV crosslinking [21, 49], filter-binding assays [22, 30, 41], electrophoretic mobility shift assays (EMSA) [26, 36, 40, 42, 47, 54], and agarose electrophoretic mobility shift assays (AGESA) [17, 41]. Each of these methods has its unique experimental advantages [55]. Acknowledging that binding between a nucleic acid and RNA-binding protein (RBP) can be affected by differences in posttranslational modification [56] and/or differences between different protein variants [57, 58] our working hypothesis is that given a particular RBP, a particular RNA and a defined buffer each of these methods should converge to produce a common answer. While extensively studied, FMRP's interactions with RNA have not always been examined with this hypothesis in mind Table 1, and it has been suggested that differences in experimental conditions and protein preparations form the basis for the divergent results obtained for the FMRP BC1 RNA interaction [59, 60].

**Figure 1:**
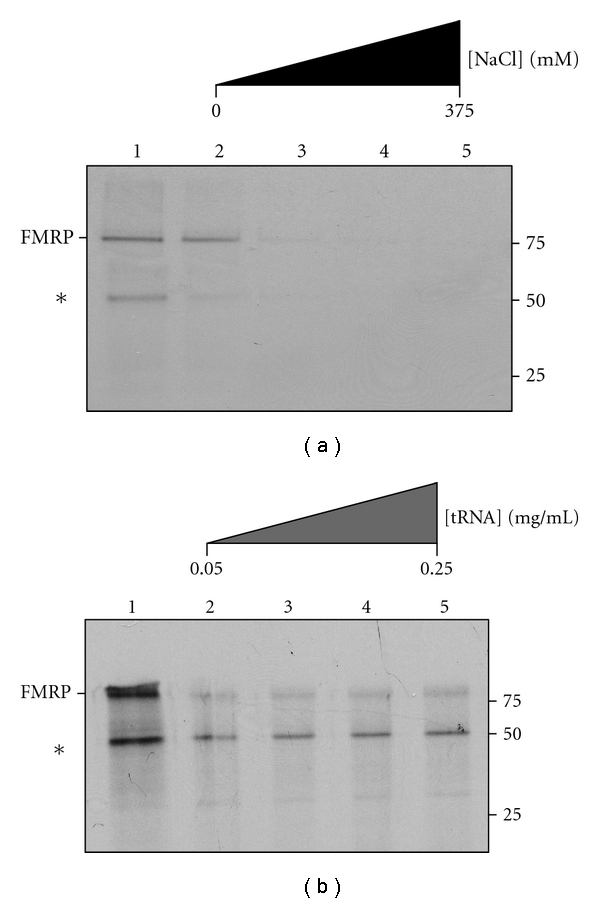
NaCl blocks nonspecific binding of FMRP with avidin. (a) ^35^S-FMRP was bound to SoftLink resin in 1x Schaeffer binding buffer supplemented with 0 mM, 125 mM, 250 mM and 375 mM NaCl, lanes 2–5, respectively. Bound ^35^S-FMRP was recovered, resolved by SDS-PAGE and subject to autoradiography. Lane 1 shows the amount of ^35^S-FMRP input into the assay. (b) ^35^S-FMRP was bound to SoftLink resin in 1x Schaeffer binding buffer supplemented with 0.05 mg/mL, 0.1 mg/mL, 0.15 mg/mL, and 0.25 mg/mL tRNA, lanes 2–5, respectively. Bound ^35^S-FMRP was recovered, resolved by SDS-PAGE and subject to autoradiography. Lane 1 shows the amount of ^35^S-FMRP input into the assay. The asterisk marks a ^35^S-truncation product produced by transcription/translation.

We chose to examine heretofore unstudied aspects of FMRP's interaction with BC1 RNA-using affinity capture, one of the older techniques used in FMRP RNA binding studies. We next sought a basic buffer to use. In several previous publications, we have used a buffer described by Schaeffer et al. [26], which used physiological saline [17, 41, 47], Table 1. However, this buffer contains tRNA to reduce nonspecific binding, and as two publications demonstrate that FMRP can interact with tRNA [42, 47], we first determined whether specific binding between FMRP and RNA could be observed without the addition of tRNA. As shown in Figure 1 (upper panel), in the presence of physiological salt and in the absence of RNA, ^35^S-FMRP produced by *in vitro* translation in rabbit reticulocyte lysate (RRL) nonspecifically bound to the avidin affinity column; however, addition of increasing amounts of NaCl decreased this nonspecific interaction so that at 125 mM NaCl the amount of bound FMRP was between 5%–10% of the total. As expected, tRNA also blocked the association of full-length FMRP to the avidin column, Figure 1 (lower panel). Thus, Schaeffer buffer supplemented with 125 mM NaCl blocks the non-specific interaction of FMRP with the affinity matrix as effectively as Schaeffer buffer supplemented with tRNA.

### 3.2. Prior Annealing of BC1 RNA Enhances Its Interaction with FMRP

**Figure 2:**
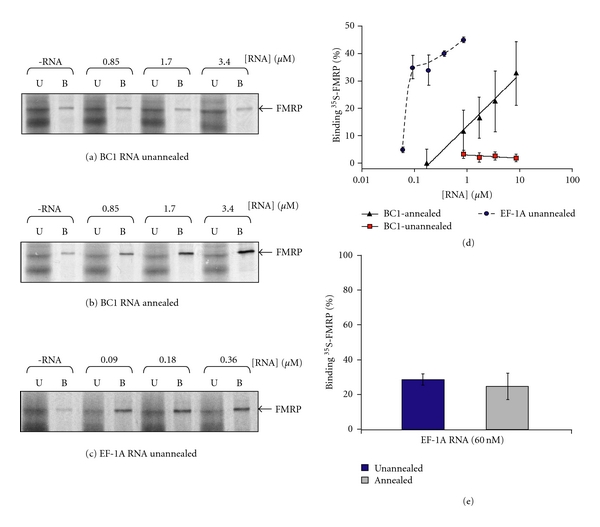
FMRP interacts weakly with annealed BC1 RNA. Equal amounts of ^35^S-FMRP was titrated with various amounts of (a) unannealed biotinylated-BC1 RNA, (b) annealed biotinylated-BC1 RNA, and (c) unannealed biotinylated-eEF-1A RNA, as indicated. Bound (B) and unbound (U) ^35^S-FMRP was recovered, resolved by SDS-PAGE and subject to autoradiography. Nonspecific binding to the SoftLink avidin resin is shown in the -RNA lanes. (d) Graphical analysis of the above data for 4 reactions per concentration. (e) Binding of annealed or unannealed eEF1A RNA (60 nM) to ^35^S-FMRP. The percent binding corrected for background of 4 reactions per RNA type is plotted (*P* = .45 by ANOVA).

Having established this basic set of conditions, we applied them to study the FMRP BC1 RNA interaction. The weak binding between FMRP and BC1 RNA that occurs in protein excess [36] suggested that the protein might be surveying the population of BC1 RNA conformers and interacting with a particular one. As BC1 RNA is known to form higher order structure [45], we performed a simple experiment to perturb the conformer population that would test this hypothesis. BC1 RNA was transcribed *in vitro* and purified by salt and alcohol precipitation; then, it was either heated briefly or left untreated. Each RNA (annealed and unannealed, resp.) was then was titrated with a constant amount of ^35^S-FMRP, and the binding was assessed by affinity capture. Under the conditions used, unannealed BC1 RNA bound extremely weakly at all concentrations examined, in concert with previous obtained results [41, 64]. On the other hand, annealed BC1 RNA exhibited stronger binding to FMRP over the range of concentrations examined, Figures  2(a) and 2(b).

To index the above results to a known standard, the interaction between eEF-1A mRNA and FMRP was also measured in parallel. Previous work had shown that this RNA binds strongly to FMRP, without prior annealing [17, 41]. As expected, the FMRP eEF-1A mRNA interaction was much stronger than the FMRP BC1 RNA interaction, Figure  2(c). In fact, with the same amount of ^35^S-FMRP, unannealed eEF-1A mRNA evinced saturable binding at 90 nM RNA, while it took 100-fold more annealed BC1 RNA to achieve comparable binding; see Figure  2(d).

To determine whether annealing affected the interaction of FMRP with eEF-1A mRNA, binding to annealed and unannealed forms of the message was assessed using a subsaturating concentration of eEF-1A RNA. As shown in Figure  2(e), binding of eEF-1A mRNA to ^35^S-FMRP was not markedly affected by annealing.

### 3.3. Annealing Alters the Structure of BC1 RNA

**Figure 3:**
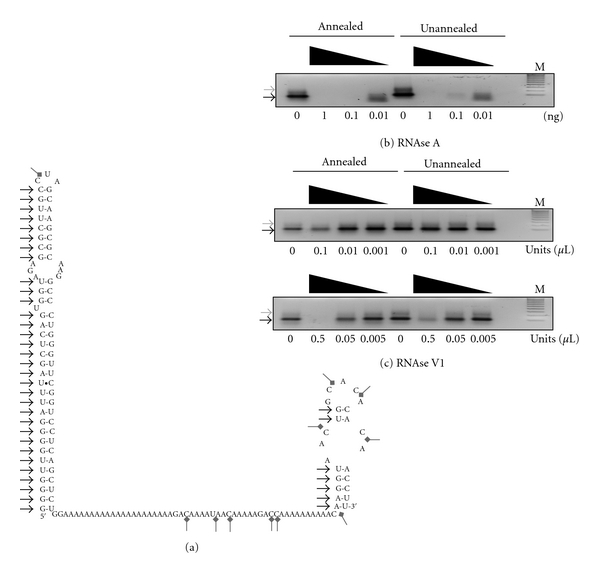
Annealed BC1 RNA and unannealed BC1 RNA differ structurally. (a) Secondary structure model of BC1 RNA from M-fold. Black arrows indicate potential RNase VI cleavage sites (both sides of the stem); gray arrows mark potential RNase A cleavage sites. (b) Serial treatment of annealed and unannealed full-length BC1 RNA with RNase A starting at 1 ng as indicated. (c) Ten-fold serial treatment of annealed and unannealed full-length BC1 RNA with RNase VI starting with 0.1 units/*μ*L (upper panel), or 0.5 units/*μ*L (lower panel) as indicated. RNA was visualized by ethidium bromide staining. Arrows mark the major conformers that can be resolved in this system. Lane M shows 100 bp molecular weight markers.

The above data implied that the conformer populations of annealed and unannealed BC1 RNA differ. To determine whether this was so, the annealed and unannealed BC1 RNAs used in Figure 2 were treated with RNases whose cleavage depends on known RNA structural features and the products were resolved on nondenaturing agarose gels. In the absence of treatment, both RNAs displayed a major band as well as a less prominent, slower migrating band, Figure 3(b). These data indicate that annealed BC1 RNA and unannealed BC1 RNA contained multiple conformers. Interestingly, in the presence of RNase A, which preferentially cleaves at single-stranded C and U residues, both annealed and unannealed BC1 RNA behaved nearly identically and were completely degraded by all but the lowest amount of the enzyme. However, when the RNAs were treated with a range of concentrations of RNase V1 (0.01–1 units), which cleaves base-paired nucleotides, the unannealed form was refractory to cleavage, while the annealed form was sensitive toward cleavage at the highest amount, Figure 3(c) (upper panel). Increasing the amount of RNase V1 recapitulated the sensitivity of annealed BC1 RNA, Figure 3(c) (lower panel); however, it also demonstrated that a fraction of unannealed BC1 RNA also contained RNase V1-sensitive stable duplex RNA. To determine whether the unique BC1 RNA conformer(s) produced by annealing could be stabilized by Mg^+2^ the annealing reaction was also carried out in the presence of 2 mM MgCl_2_. The results indicated that the magnesium did not alter the distribution of BC1 RNA conformer(s) or affect their resistance to RNase V1 (not shown).

These data unequivocally demonstrate that the conformer populations of annealed and unannealed BC1 RNA differ, and this difference is due to an increase in the amount of stable RNA duplexes in the annealed RNA. Moreover, the data support the hypothesis that the binding between FMRP and annealed BC1 RNA results from a unique conformer that is absent from the unannealed BC1 RNA conformer population.

### 3.4. Functional Dissection of the FMRP BC1 RNA Interaction

**Figure 4:**
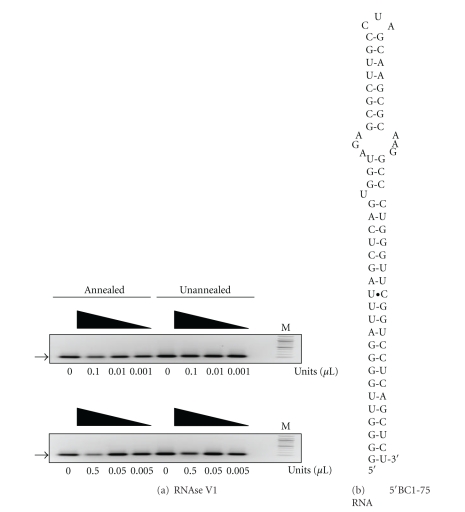
Functional dissection of structural elements in the 5′ end of BC1 RNA. (a) Ten-fold serial treatment of annealed and unannealed 5′BC1-75 RNA with RNase VI starting at 0.1 units/*μ*L (upper panel) or 0.5 units/*μ*L (lower panel). Arrow marks the major conformer. Lane M shows 100 bp molecular weight markers. (b) Secondary structure model of 5′BC1-75 RNA from M-fold.

**Figure 5:**
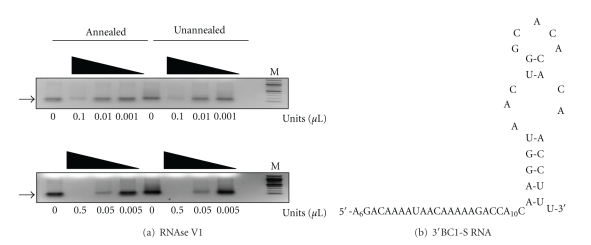
Functional dissection of structural elements in the 3′ end of BC1 RNA. (a) Ten-fold serial treatment of annealed and unannealed 3′BC1-60 RNA with RNase VI starting at 0.1 units/*μ*L (upper panel) or 0.5 units/*μ*L (lower panel). Arrow marks the major conformer. Lane M shows 100 bp molecular weight markers. (b) Secondary structure model of 3′BC1-60 RNA from M-fold.

BC1 RNA contains several distinct higher order structural elements. Its 5′ end contains two cis-acting spatial targeting elements, DTE1 and DTE2 [45]. The former is required for somatic export of BC1 RNA into dendrites, while the latter specifies long-range distal delivery and is mediated by a prominent GA-type kink turn (KT) motif in the apical region of the 5′ BC1 domain [45]. The 3′ 60 bases of BC1 RNA contains an abbreviated A-rich region and a 3′-terminal stem-loop structure [65] that has been shown to bind synergistically to the eucaryotic initiation factor 4A (eIF4A) and the polyadenylation binding protein (PABP) [41]. To further probe this interaction, BC1 RNA was functionally dissected, and these two elements were examined individually. As shown in Figure 4(a), RNase VI treatment of the 5′ 75 bases of BC1 RNA recapitulated the results of full-length BC1 RNA indicating that it forms a stable secondary structure in the absence of the 3′ end. This agrees with modeling studies using the Zuker M-fold algorithm, which show that the first 75 bases of BC1 RNA fold identically to that portion of the full-length molecule, Figure 4(b). On the other hand, annealing had no effect on the sensitivity of the 3′ 60 bases of BC1 RNA towards RNase VI, Figure 5. Thus, these data demonstrate that the difference in the conformer populations of annealed and unannealed BC1 RNA most likely arise from alterations in the 5′ end of the molecule.

**Figure 6:**
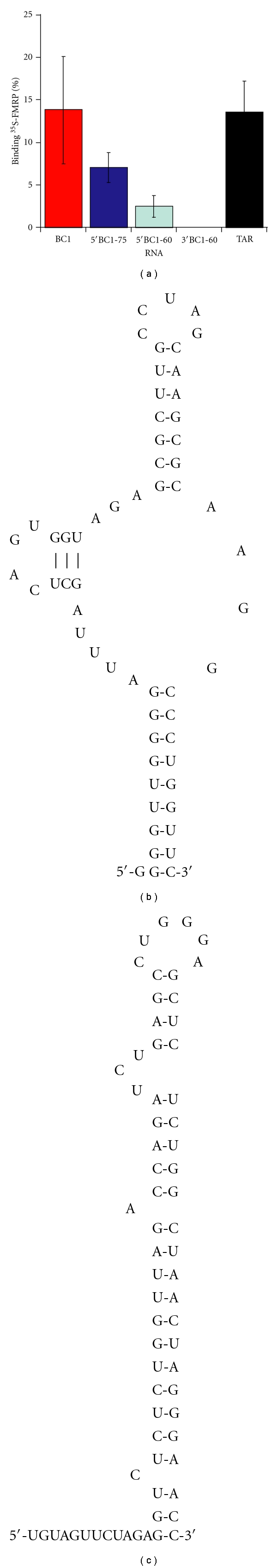
Interaction of FMRP with the 5′ and 3′ ends of BC1 RNA. (a) ^35^S-FMRP was incubated with 2 *μ*M annealed full-length BC1 RNA, or annealed versions of the first 75 b of BC1 RNA (5′BC1-75), the first 60 b of BC1 RNA (5'BC1-60) the last 60 b of BC1 RNA (3′BC1-60) and an 85 b transcript containing the HIV1 TAR hairpin. The percent binding corrected for background of 6 reactions per protein is plotted. (b) Secondary structure model of 5′BC1-60 RNA from M-fold. (c) Secondary structure model of the HIV1 TAR RNA from M-fold; the 27 b leader sequence has no effect upon the folding.

We next examined whether FMRP preferentially bound either of the dissected RNAs. Therefore, ^35^S-FMRP was incubated with 2 *μ*M annealed versions of each RNA; binding was subsequently assessed by affinity capture and compared to that of annealed full-length BC1 RNA. We found that the 5′ 75 bases of BC1 RNA bound slightly less, than full-length BC1 RNA, Figure 6(a); however, the difference did not rise to the level of statistical significance (*P* = .15, ANOVA). Truncation of this RNA by a 15 base deletion at its 3′ end further decreased binding (*P* = .08, ANOVA). Significantly, this 60 base RNA is not expected to recapitulate the folding of the first 60 bases of full-length BC1 RNA, Figure 6(b). On the other hand, the 3′ 60 bases of BC1 RNA evinced no evidence of binding, Figure 6(a). These data suggest that the major determinant that FMRP recognizes in annealed BC1 RNA is the hairpin structure of the 5′ end. To test this further, we also conducted binding studies using an 85 base RNA harboring the HIV1 TAR hairpin, Figure 6(c). The results show that this RNA binds with the same affinity as annealed full-length BC1 RNA.

### 3.5. Specificity and Requirements of the FMRP BC1 RNA Interaction

**Figure 7:**
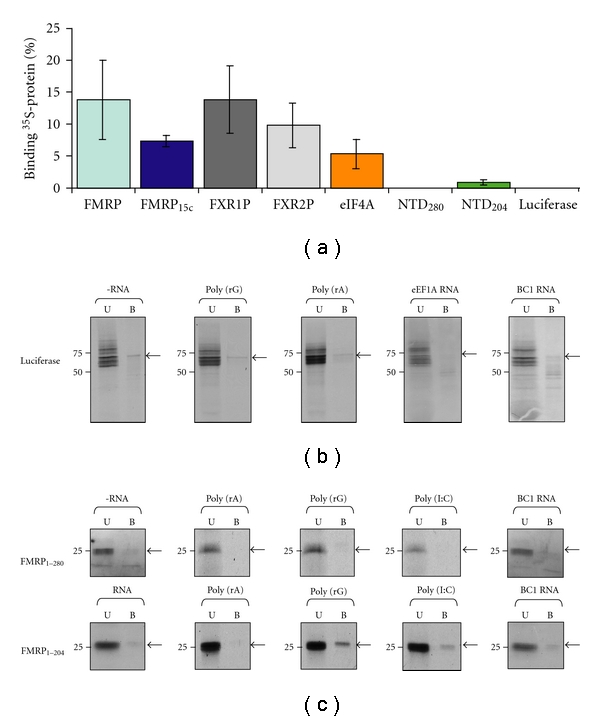
Annealed BC1 RNA binds pleiotropically to the FXRPs. (a) Equimolar amounts of ^35^S-FMRP, ^35^S-FMRP_15c_,^ 35^S-FXR1P, ^35^S-FXR2P, ^35^S-eIF4A, ^35^S-FMRP_280_, ^35^S-FMRP_204_, and ^35^S-Luciferase were incubated with 2 *μ*M annealed BC1 RNA. The percent binding corrected for background of 6 reactions per protein is plotted. The relative levels of FMRP and FMRP_15c_, FMRP and FXR1P and FMRP and FXR2P were not significantly different (*P* = .17, *P* = .67 and *P* = .27, resp., ANOVA). However, the relative levels of FMRP and eIF4A and FMRP and NTD_204_ were significantly different (*P* = .038 and *P* = .007, resp., ANOVA). Since no binding above background was observed for NTD_280_ and Luciferase relative differences were not assessed. (b) Pull-down assays between ^35^S-luciferase and poly (rG), poly (rA), eEF1A RNA (90 nM) and annealed BC1 RNA (5 *μ*M) showing the unbound (U) and bound (B) fractions. Binding in the absence of RNA is shown for the negative control. (c) Pull-down assays between ^35^S-NTD_280_ or ^35^S-NTD_204_ and poly (rG), poly (rA), poly (rI : rC) and annealed BC1 RNA (5 *μ*M) showing the unbound (U) and bound (B) fractions. Binding in the absence of RNA is shown for the negative control.

Different preparations or sources of FMRP has been posited as a potential explanation for the divergent BC1 RNA-binding data obtained by different laboratories [59, 60] and a likely cause of such differences is in the posttranslation modifications that occur in each. To begin to address this question, we examined whether differences in posttranslational arginine methylation could alter the binding affinity of annealed BC1 RNA and FMRP. Specifically, we compared the ability of full-length FMRP and the ability of an alternatively spliced variant FMRP_Ex15c_ to bind 2 *μ*M annealed BC1 RNA. Previous studies showed that full-length FMRP is readily methylated in its RG-rich region, while FMRP_15c_, which lacks 25 amino acids upstream of the RG-rich region, is refractory to arginine methylation [66].  Figure 7(a) shows that binding of annealed BC1 RNA to FMRP_15c_ was less than it was to full-length FMRP, but the difference did not rise to statistical significance.

We next examined whether the binding between annealed BC1 RNA and FMRP was unique. As an initial indicator, 2 *μ*M annealed BC1 RNA was incubated with equimolar amounts of fragile X family members, ^35^S-FMRP, ^35^S-FXR1P and ^35^S-FXR2P (full-length forms); subsequently, binding was assessed as previously described. As shown in Figure 7(a), both FXR1P and FXR2P bound as well as FMRP. To confirm and extend these results binding studies between annealed BC1 RNA and luciferase or eIF4A were performed. Luciferase does not contain any RNA-binding motifs and does not bind to poly (rA), poly (rG), or eEF-1A mRNA, Figure 7(b). Correspondingly, we found that luciferase did not interact with annealed BC1 RNA. On the other hand, the RNA helicase, eIF4A, bound weakly annealed BC1 RNA under these conditions, Figure 7(a). These data indicate that in addition to FMRP annealed BC1 RNA can interact interchangeably with at least three other RNA-binding proteins.

It has been previously proposed that BC1 RNA interacts with the N-terminal domain (NTD) of FMRP [40]. Nevertheless, Wang et al. did not observe an interaction between unannealed BC1 RNA and a 280 amino acid NTD construct, FMRP_1–280_ [41]. To complete this analysis, we also assessed the interaction of annealed BC1 RNA with FMRP_1–280_. As shown in Figure 7(a), annealed BC1 RNA did not bind to ^35^S-FMRP_1–280_. To determine whether ^35^S-FMRP_1-280_ lacked the ability to interact with RNA, we asked whether it could to bind to homoribopolymers. As shown in Figure 7(c), ^35^S-FMRP_1–280_ was unable to bind to poly(rA), poly(rG), or poly(rI : rC). These data suggest that ^35^S-FMRP_1–280_ does not significantly interact with ribonucleic acids under the conditions used. Interestingly, however, while a shorter NTD, that is, ^35^S-FMRP_1–204_, was also unable to bind to annealed BC1 RNA, Figure 7(a), it was able to bind specifically to poly(rG) and to a lesser extent, poly(rI : rC), Figure 7(c). Thus, annealed BC1 RNA does not interact significantly with the 204 N-terminal residues of FMRP.

## 4. Conclusions

Disparate data obtained using electrophoretic mobility shift assays have been published concerning the binding of FMRP to BC1 RNA [36, 40, 47]. To investigate this discrepancy, we turned to an affinity capture assay to measure RNA binding to FMRP under the assumption that the results from different assays should converge to produce a common result. This particular assay was first described by Boehlens et al. and applied to the interactions of FMRP and nucleic acids by Ashley et al. [51, 67]. Here, we demonstrate that in 150 mM KCl and 125 mM NaCl, the assay has a dynamic range of RNA binding of at least two logs. Thus, it is able to distinguish high affinity binding from low affinity binding. 

We specifically developed these assay conditions, which do not utilize tRNA as a general nonspecific binding competitor, because it had previously been demonstrated that both tRNA and BC1 RNA bind to FMRP in a low salt buffer with nearly identical dissociation constants (K_d_s) [42], and because tRNA was shown to directly displace BC1 RNA from FMRP [47]. Thus, tRNA, while admittedly effective, may not be the best blocking reagent for examining FMRP's interaction with BC1 RNA. On the other hand, FMRP's association with polyribosomes [37, 38], translationally repressed ribosomes within neuronal granules [68], and its function in translational regulation [17, 19, 27] indicates that it operates in an environment with a relatively high local concentration of tRNA, which would ensure a direct competition with BC1 RNA. Indeed, previously published immunoprecipitation experiments, which are carried out in the presence of a large excess of tRNA (both added and from the endogenous tRNA present during cell lysis) and which fail to find BC1 RNA among the precipitated transcripts are entirely consistent with a direct competition between BC1 RNA and tRNA [47]. Here, we demonstrate that BC1 RNA produced simply by in vitro transcription does not interact with FMRP even in the absence of tRNA, confirming our previous results using different assays and different binding conditions [41]. Nevertheless, by artificially annealing the BC1 RNA transcript, we found that binding occurred although it was at least a hundred-fold weaker than that between FMRP and eEF-1A mRNA. Previous protocols examining the interaction between BC1 RNA and FMRP have not indicated that the RNA was pretreated or annealed before binding to FMRP was initiated [36, 40, 47]; therefore, it must be assumed that it was not, and hence, this treatment is unique to this work.

To try to understand the result, we first determined whether enhanced binding due to annealing was a general feature of FMRP's interaction with RNA. A search of the current literature produced mixed results. Darnell et al., working with kissing complex RNA a small double hairpin with additional loop-loop tertiary interactions that binds to FMRP's KH_2_ domain, preannealed the RNA before binding was initiated [30]. Likewise, Ramos et al. and Zanotti et al. preannealed the synthetic G-rich RNA, sc1, to form G-quartets before binding to RGG box peptides [69, 70]. Finally, Bechara et al. renatured SoSLIP RNA, a small multiple hairpin-containing RNA, before they used it in binding reactions with FMRP [19]. In contrast, longer RNAs containing U-rich motifs such as MBP mRNA [71], the FMR1 3′UTR [21], eEF-1A mRNA [17], and BMP receptor mRNA [41] do not require preannealing to bind tightly to FMRP. However, none of these studies directly compare binding between annealed and unannealed RNAs. Therefore, we examined the effect annealing eEF-1A mRNA had on its binding to FMRP. Under conditions where FMRP's interaction with unannealed eEF-1A was linearly related to the input RNA we found that preannealing the RNA had no measurable effect upon the binding. Thus, while not a general requirement for FMRP binding preannealing may be necessary for smaller RNAs whose structure is more susceptible to changes in temperature, buffer pH, concentration and type of ions and RNA concentration [70]. 

We next determined whether annealed BC1 RNA was structurally altered from unannealed BC1 RNA. Indeed, we showed that the annealed form exhibited an increased susceptibility to cleavage by RNase VI. These data support the hypothesis that a particular BC1 RNA conformer; that is, one containing a unique doubled-stranded RNA structure was the true FMRP interactor. Functional dissection of BC1 RNA into either a 5′ ID element or a 3′ element demonstrated that the major determinant that FMRP binds to is located in the 5′ 75 bases of the molecule. As FMRP also binds to tRNA and TAR RNA, these data imply that FMRP can weakly recognize hairpin-containing RNAs. 

Another difference between the published FMRP BC1 RNA interaction studies was that one group used full-length recombinant FMRP produced from baculovirus [36, 40], while the other group used full-length recombinant FMRP produced in *E. coli *[47]. A potential concern here is that each of these preparations may be differentially posttranslationally modified. Indeed, it is well known that FMRP is subject to posttranslational methylation of arginine residues in its RG-rich region [18, 31]. However, while protein arginine methyltransferases are present in *Spodoptera frugiperda* [72], the host cells used in baculovirus FMRP production [40], they are absent from *E. coli* [73, 74]. Thus, it is possible that the presence or absence of methyl-arginine residues might directly or indirectly affect an interaction between FMRP and BC1 RNA. In the present work, we opted to produce full-length FMRP in rabbit reticulocyte lysates (RRL), which generates partially methylated FMRP [66]. Again, we assumed that posttranslational modifications arising from source differences could be a modulating but not *the* determining factor in a putative FMRP BC1 RNA interaction. Precedence for this assumption may be seen in the work of Stetler et al. who showed that methylating FMRP decreased, but did not abrogate, the direct binding of two G-quartet RNAs to FMRP's RG-rich region [56]. Consistent with the work using *E. coli*-purified FMRP [47] and with the work of Wang et al. who used unmethylated FMRP produced in cell-free wheat germ lysates to examine BC1 RNA binding [41], we observed minimal interactions between RRL-produced FMRP and unannealed BC1 RNA. Thus, methylating FMRP's arginine residues does not enhance its affinity for unannealed BC1 RNA. Furthermore, although binding between annealed BC1 RNA and the methylation refractory form of FMRP (FMRP_15c_) was reduced compared to methylated FMRP, it was not a statistically significant reduction. Thus, methylarginine residues in FMRP's RG-rich region *are not absolutely required* for this interaction either. 

We also probed the exclusivity of FMRP's interaction with annealed BC1 RNA. Here, we found that interactions of similar magnitude occur with fragile X paralogs FXR1P and FXR2P and to a lesser extent, eIF4A. These results demonstrate that BC1 RNA binds indiscriminately to RNA-binding proteins. Interestingly, contrasting results were recently obtained for kissing complex (kc) RNA [34], where it was shown that this RNA exclusively interacts with the fragile X paralogs but not with other related KH domain-containing RNA-binding proteins. 

As all three paralogs have highly homologous N-terminal sequences but are more divergent in their C-terminal ends [75], we inquired whether annealed BC1 RNA interacted with specific N-terminal fragments of FMRP (NTDs). We did not observe an interaction between annealed BC1 RNA and FMRP_1–280_, a construct comprising both N-terminal residues and the first KH domain (KH_1_). The results were consistent with previous work showing that this construct did not interact with unannealed BC1 RNA [41]. However, as FMRP_1–280_ did not interact with other nucleic acids under these conditions, we could not rule out the possibility that the protein was mis-folded although previous physical evidence suggests this is not the case [50]. Therefore, we also examined the interaction between annealed BC1 RNA and FMRP_1–204_, a construct that does interact with nucleic acids and is expected “to be at least partially folded and be monodisperse” under the conditions it was used [50]. Nevertheless, annealed BC1 RNA did not interact with this FMRP fragment either. Hence, the first 204 residues of FMRP, which includes two Tudor domains and an alpha helix that is essential for domain stability [76], are not required for the interaction of annealed BC1 RNA with FMRP. Using different conditions, Zalfa et al. have suggested that BC1 RNA binds to an NTD comprising amino acids 1–217 [40]. The additional 13 residues of this construct comprise part of another alpha helix, which stabilizes the folding of FMRP's KH_1_ domain [50]. While it is possible that amino acids 205–217 may confer binding specificity to annealed BC1 RNA, Zalfa et al. showed that a construct comprising this alpha helix along with the KH_1_ domain (residues 205–280) did not interact with BC1 RNA [40]. However, additional studies using other constructs will be needed to fully address the question of which FMRP residues are necessary and sufficient for its interaction with annealed BC1 RNA.

Although we demonstrate an in vitro interaction between FMRP and BC1 RNA, its nature and its physiological significance remain elusive. For example, the structure of the unique BC1 RNA conformer that FMRP binds to has not been defined other than the fact that it contains double-stranded RNA and is primarily found in the 5′ half of the molecule. In fact, it is possible based on the rather high RNA concentrations used in the annealing reaction that FMRP may be interacting with a dimer of BC1 RNA. This would be analogous to the formation of intermolecular G-quartet RNAs such as MAP1B RNA [69]. Clearly, a more comprehensive biophysical analysis of the parameters of this interaction is needed to fully address this issue. Regardless, the ultimate significance of this observation is questionable given that the formation of this conformer does not occur at temperatures that mammalian cells can survive. Although one might postulate that a chaperone protein may be able to mitigate the temperature requirement for annealing [77, 78], it is clear that FMRP cannot be this chaperone, because it is unable convert unannealed BC1 RNA into a molecule that it binds under physiological conditions. Given this, our data generated using different constructs, different preparations and different methods, converge with the published work of Iacoangeli et al. [47] to support a model in which FMRP and BC1 RNA operate independently of each other to control protein synthesis in neuronal processes.

## Figures and Tables

**Table 1:** Binding conditions used to measure the interaction between FMRP and RNA.

Publication	Assay	Conditions
Ashley et al. (1993) [51]	Pull-down	16 mM HEPES-KOH pH 7.9, 120 mM KCI, 0.04% Nonidet P-40, 1 mg/mL BSA, 0.16 mM dithioerythritol, 0.4 mM phenylmethylsulfonyl fluoride

Brown et al. (1998) [49]	Pull-down	10 mM Tris-HCl pH 7.5, 2.5 mM MgCl_2_, 100 mM NaCl, 2.5% Trition X100, 1 mg/mL heparin

Price et al. (1996) [52]	Pull-down	20 mM Hepes, pH 7.9, 2 mM MgCl_2_, 10 mM ZnCl_2_, 70 mM NH_4_Cl, 0.02% Nonidet P-40, 5 mg/mL yeast tRNA

Sung et al. (2000) [21]	Pull-down Filter-Binding	20 mM Hepes, pH 7.9, 2 mM MgCl_2_, 10 mM ZnCl_2_, 70 mM NH_4_Cl, 0.02% Nonidet P-40, 5 mg/mL yeast tRNA

Denman and Sung (2002) [57]	Pull-down	20 mM Hepes, pH 7.9, 2 mM MgCl_2_, 10 mM ZnCl_2_, 70 mM NH_4_Cl, 0.02% Nonidet P-40, 5 mg/mL yeast tRNA

Schaeffer et al. (2001) [26]	EMSA	50 mM Tris-HCl pH 7.4, 1 mM MgCl_2_, 1 mM EDTA, 150 mM KCl, 1 mM DTT, 0.25 mg/mL of *E.coli* tRNA, 0.01% BSA, 8 U of RNasin

Sung et al. (2003) [17]	Pull-down EMSA	50 mM Tris-HCl, pH 7.0, 2 mM MgCl_2_, 150 mM NaCl, 1 mM DTT, 0.25 mg/mL *E.coli* tRNA, 0.25 mg/mL BSA

Bechara et al. (2006) [61]	EMSA	50 mM Tris-HCl pH 7.4, 1 mM MgCl_2_, 1 mM EDTA, 150 mM KCl, 1 mM DTT, 0.25 mg/mL of *E.coli* tRNA, 0.01% BSA, 8 U of RNasin

Didiot et al. (2008) [54]	EMSA	50 mM Tris-HCl pH 7.4, 1 mM MgCl_2_, 1 mM EDTA, 150 mM KCl, 1 mM DTT, 0.25 mg/mL of *E. coli* tRNA, 0.01% BSA, 8 U of RNasin

Zalfa et al. (2003) [36]	EMSA	10 mM HEPES pH 7.9, 3 mM MgCl_2_, 10 mM DTT, 100 mM KCl, 750 mM NaCl, 5% glycerol, 7 mM *β*-Mercaptoethanol, 1 mg/mL Albumin, 1.3 mg/mL Heparin

Zalfa et al. (2005) [40]	EMSA	20 mM HEPES-KOH, pH 7.6, 5 mM MgCl_2_, 300 mM KCl, 2 mM DTT, 5% glycerol, and 500 ng of total yeast tRNA or 20 *μ*g of heparin.

Darnell et al. (2001) [22]	Filter-Binding	10 mM Tris-OAc pH 7.7, 200 mM KOAc, 5 mM MgOAc_2_

Darnell et al. (2005) [30]	Filter-Binding	50 mM Tris-OAc at pH 7.7, 50 mM KOAc, 10 mM DTT, 5 mM Mg(OAc)_2_, 30 *μ*g/mL tRNA

Gabus et al. (2004) [42]	EMSA	20 mM Tris-HCl pH 7.5, 30 mM NaCl, 0.2 mM MgCl_2_, 5 mM DTT, 10 *μ*M ZnCl_2_

Laggerbauer et al. (2001) [16]	Pull-down	PBS, 0.02% IGEPAL, 1% BSA

Siomi et al. (1993) [15]	Pulldown	10 mM Tris-HCl pH 7.4, 2.5 mM MgCl_2_, 0.5% Triton X-100, 100–1000 mM NaCl

Stetler et al. (2005) [56]	Pulldown	2 M KOAc, 100 mM Tris-OAc pH 7.7 and 50 mM MgOAc_2_, 1 *μ*L of yeast tRNA, 1 *μ*L of RNAsin

Menon and Mihailescu (2007) [62]	EMSA	50 mM Tris-HCl pH 7.5, 150 mM NaCl and protease inhibitors

Fahling et al. (2009) [20]	EMSA	10 mM Hepes pH 7.2, 3 mM MgCl_2_, 5% glycerol, 1 mM DTT, 150 mM KCl, 2 U/*μ*L RNaseOUT, 0.5 *μ*g/*μ*L rabbit rRNA

Zou et al. (2008) [63]	AGESA	20 mM Tris-HCl pH 7.2, 150 mM NaCl

Iacoangeli et al. (2008) [47]	EMSA	50 mM Tris-HCl pH 7.6, 150 mM KCl, 1 mM MgCl_2_, 1 mM EDTA, 1 mM DTT, 0.2 U/*μ*L RNase inhibitor, 100 ng/*μ*L total yeast tRNA, and 100 ng/*μ*L BSA

Sets of binding conditions are grouped by the different laboratories that used them. Each set of conditions is differentiated from the next by a dotted line. In some instances the same group used multiple sets of binding conditions in multiple publications.

## Abbreviations

AGESA: Agarose electrophoretic mobility shift assays
BC1 RNA: Brain cytoplasmic RNA
eEF-1A: Eucaryotic elongation factor 1A
eIF4A: Eucaryotic initiation factor 4A
EMSA: Electrophoretic mobility shift assays
FMRP: Fragile X mental retardation protein
NTD: N-terminal domain
PABP: Polyadenylation binding protein
RBP: RNA binding protein
RRL: Rabbit reticulocyte lysate.
